# Ectoderm-derived frontal bone mesenchymal stem cells promote traumatic brain injury recovery by alleviating neuroinflammation and glutamate excitotoxicity partially via FGF1

**DOI:** 10.1186/s13287-022-03032-6

**Published:** 2022-07-26

**Authors:** Qiaozhen Qin, Ting Wang, Zhenhua Xu, Shuirong Liu, Heyang Zhang, Zhangzhen Du, Jianing Wang, Yadi Wang, Zhenning Wang, Shanshan Yuan, Jiamei Wu, Wenyan He, Changzhen Wang, Xinlong Yan, Yan Wang, Xiaoxia Jiang

**Affiliations:** 1 Beijing Institute of Basic Medical Sciences, 27 Taiping Road, Haidian District, Beijing, 100850 People’s Republic of China; 2 Faculty of Environmental and Life Sciences, Beijing University of Technology, Beijing, 100124 People’s Republic of China; 3 Anhui Medical University, Hefei, 230032 Anhui People’s Republic of China; 4 Beijing Institute of Radiation Medicine, 27 Taiping Road, Haidian District, Beijing, 100850 People’s Republic of China; 5 China National Clinical Research Center for Neurological Diseases, Jing-Jin Center for Neuroinflammation, Beijing Tiantan Hospital, Capital Medical University, Beijing, People’s Republic of China

**Keywords:** Frontal bone mesenchymal stem cells, Traumatic brain injury, Neuroinflammation, Glutamate excitotoxicity

## Abstract

**Background:**

Traumatic brain injury (TBI) leads to cell and tissue impairment, as well as functional deficits. Stem cells promote structural and functional recovery and thus are considered as a promising therapy for various nerve injuries. Here, we aimed to investigate the role of ectoderm-derived frontal bone mesenchymal stem cells (FbMSCs) in promoting cerebral repair and functional recovery in a murine TBI model.

**Methods:**

A murine TBI model was established by injuring C57BL/6 N mice with moderate-controlled cortical impact to evaluate the extent of brain damage and behavioral deficits. Ectoderm-derived FbMSCs were isolated from the frontal bone and their characteristics were assessed using multiple differentiation assays, flow cytometry and microarray analysis. Brain repairment and functional recovery were analyzed at different days post-injury with or without FbMSC application. Behavioral tests were performed to assess learning and memory improvements. RNA sequencing analysis, immunofluorescence staining, and quantitative reverse-transcription polymerase chain reaction (qRT-PCR) were used to examine inflammation reaction and neural regeneration. In vitro co-culture analysis and quantification of glutamate transportation were carried out to explore the possible mechanism of neurogenesis and functional recovery promoted by FbMSCs.

**Results:**

Ectoderm-derived FbMSCs showed fibroblast like morphology and osteogenic differentiation capacity. FbMSCs were CD105, CD29 positive and CD45, CD31 negative. Different from mesoderm-derived MSCs, FbMSCs expressed the ectoderm-specific transcription factor Tfap2β. TBI mice showed impaired learning and memory deficits. Microglia and astrocyte activation, as well as neural damage, were significantly increased post-injury. FbMSC application ameliorated the behavioral deficits of TBI mice and promoted neural regeneration. RNA sequencing analysis showed that signal pathways related to inflammation decreased, whereas those related to neural activation increased. Immunofluorescence staining and qRT-PCR data revealed that microglial activation and astrocyte polarization to the A1 phenotype were suppressed by FbMSC application. In addition, FGF1 secreted from FbMSCs enhanced glutamate transportation by astrocytes and alleviated the cytotoxic effect of excessive glutamate on neurons.

**Conclusions:**

Ectoderm-derived FbMSC application significantly alleviated neuroinflammation, brain injury, and excitatory toxicity to neurons, improved cognition and behavioral deficits in TBI mice. Therefore, ectoderm-derived FbMSCs could be ideal therapeutic candidates for TBI which mostly affect cells from the same embryonic origins as FbMSCs.

**Supplementary Information:**

The online version contains supplementary material available at 10.1186/s13287-022-03032-6.

## Background

Traumatic brain injury (TBI) affects millions of individuals worldwide [1]. Although the death rate of TBI has decreased, a growing living population suffers from severe motor, neural psychological, and cognitive disabilities directly related to TBI pathogenesis [2]. External trauma might cause structural damage, oxidative stress, excessive excitatory neurotransmitters [3], and an inflammatory cascade responsible for further cell death [4], leading to anatomical and functional brain damage. Despite extensive research, there is still no ideal way for TBI therapy owing to multiple complications during pathogenesis.

Stem cell therapy have provided an ideal therapeutic option for treating TBI based on its ability to inhibit neuroinflammation, mitigate secondary cell loss and promote neurogenesis and functional recovery [5]. Neural stem cells (NSCs) and mesenchymal stem cells (MSCs) have received a great amount of attention in TBI therapy, avoiding the ethical issues surrounding the use of human embryonic stem cells and the controversial issues around induced pluripotent stem cells [6, 7]. Neural cells, astrocytes, and oligodendrocytes are derived from NSCs. Exogenous NSC transplantation not only promote the proliferation and differentiation of endogenous NSCs or neural progenitor cells (NPCs) and also differentiate into functional neurons, replacing lost neurons [8]. Additionally, grafted NSCs promote the switch of microglial cells into an anti-inflammatory phenotype, which might regulate the microenvironment, accelerate neuroprotection, enhance hippocampal neurogenesis, improve anatomical and functional outcomes [9].

MSCs are immune-privileged and display a robust ability to modulate the inflammatory response. MSC transplantation for TBI therapy has been investigated in animal models and clinical trials, showing significant improvements in neurological function [10]. Previous studies have revealed that neuro-restoration and not neuro-replacement, contributes to MSC transplantation-induced TBI recovery [11]. MSCs could secret anti-inflammatory factors (i.e., PEG2, NO, and IL-10) [11–13] and growth factors (i.e., VEGF, HGF, and BDNF) [14–16] that suppress the inflammatory cascade, enhance neurogenesis, and promote synaptogenesis. MSCs can be isolated from a multitude of adult tissues, such as bone marrow, umbilical cord, adipose tissues, which are mesoderm-derived and such as cranial, frontal bone tissues, which are ectoderm-derived [17]. Previous studies have proposed that damaged tissue from different germ layers will recruit MSCs from corresponding germ layers for repair [18, 19]. Moreover, cells derived from the same lineage are more effective in grafting procedures and promoting injury recovery [20]. The primary events following TBI include neuroinflammation, oxidative stress, excitotoxicity, and cell death [21]. Regulatory factors from MSCs such as exosomes, IL-10, IL-4 were shown to control neuroinflammation and promote a change from destructive inflammatory processes to neuroprotective [22–24]. Whether these ectodermal lineage-derived MSCs will secret some modulatory factors and be good candidates for the therapy of brain injury where most damaged cells derived from ectoderm requires further investigation.

In this study, we aimed to investigate the role and underlying mechanism of ectoderm-derived frontal bone MSCs (FbMSCs) in suppressing neuroinflammation, promoting neurogenesis, and improving cognition and behavioral deficits in TBI mice. Our study helped to identify FbMSCs from the same embryonic origins as neural cells could be potential ideal candidates for TBI therapy.

## Material and methods

### Animals

Newborn or 8–10 weeks old C57BL/6 N mice were purchased from Beijing Vital River Laboratory Animal Technology (Beijing). All mice were housed in a standard animal facility under controlled temperature (21 °C) and photoperiod (12 h light/12 h dark) with free access to water and food. All animal experiments were carried out in accordance with the "guidelines for the care and use of experimental animals" approved by Beijing Institute of Basic Medical Sciences. The ethical review committee of animal experimental institutions approved all experimental protocols.

### Isolation and culture of mesenchymal stem cells

Primary bone mesenchymal stem cells (BMSCs) were isolated from murine compact bone and culture-expanded as described in our previous report [25].

Frontal bone mesenchymal stem cells (FbMSCs) and parietal bone mesenchymal stem cells (PbMSCs) were prepared from frontal or parietal bone of newborn C57BL/6 N, respectively. All the meninges were thoroughly removed from bone. Bones were carefully excised into chips and transferred in α-MEM containing 10% fetal bovine serum (FBS) with the presence of 2.5% trypsin (Biological Industries; 2,038,152). The enzyme treated bone chips were washed three times and then cultured in α-MEM supplemented with 10% FBS and 1% penicillin/streptomycin in a humidified incubator at 37 °C and 5% CO2. Culture medium was replaced every 2 days. Passage was twice per week at a split ratio of 1:2 or 1:3. Cells at passages 3–4 were used for the subsequent experiments.

FbMSCs were stimulated without or with 20 ng/mL IL6 (Pepro Tech, 216-16), 100 ng/mL LPS (Sigma, L2630), 20 ng/mL TNFα (Pepro Tech, 315-01A) plus 20 ng/mL IFNγ (Pepro Tech, 315-5), respectively, for 24 h and then collected for analysis.

### Alkaline phosphatase (ALP) staining

ALP staining was performed using a NBT/BCIP staining kit (CoWin Biotech, Beijing, China) as previously described [26]. After osteogenic induction for 7 days, cells were fixed in 4% paraformaldehyde for 10 min, and then ALP staining was performed following the manufacturer’s instructions.

### Flow cytometry analysis

FbMSCs were collected and washed twice with washing buffer. Cells were then incubated with primary antibody diluted in PBS for 30 min at 4 °C. Antibodies against CD29 (FITC, 102,205), CD105 (PE, 120,408), CD31 (PE, 102,407), and CD45 (APC, 147,707) were purchased from BioLegend. The isotype-matched mouse IgG1-FITC, IgG1-PE, and IgG1-APC were used as negative controls. Data were collected on a FACS Canto II (BD) and were analyzed with FlowJo software (TreeStar).

### Traumatic brain injury (TBI) surgery

Animals were subjected to either TBI using a controlled cortical impact (CCI) injury model or sham control (craniotomy with no injury). Before CCI injury, each animal was intraperitoneally injected with 2,2,2-Tribromoethanol (350 mg/kg, Sigma-Aldrich, T48402). Animals were placed on a stereotaxic frame attached to a temperature-controlled heating pad (37 °C) with their scalp depilated. After exposing the skull, a 4-mm craniectomy was performed over the cortex (3.0 mm AP and 2.0 mm ML to bregma). A pneumatically operated metal impactor (diameter = 3 mm) impacted the brain at a velocity of 3.5 m/s, reaching a depth of 1.0 mm below the dura mater layer, and remained in the brain for 400 ms. In Matrigel-treated TBI group and FbMSC-treated TBI group, 5 μl Matrigel (Corning, 356,234) or 5 × 10^5^ FbMSCs mixed with 5 μl Matrigel for each mouse was applied to the injury site, respectively.

### Cell co-culture

Primary cortical neurons were prepared from embryonic day 15–17 mice embryos and plated on 24 well plate pretreated with 0.1 mg/mL poly-d-lysine (Sigma, 25,988-63-0) at a density of 8 × 10^4^/cm^2^. Neurons were plated and maintained in Neurobasal medium (Gibco, A2477501) supplemented with 2% B27 and 1% GlutaMAX (Gibco, 35,050,079) in a humidified incubator at 37 °C with 5% CO2. Neurons cell line HT22 (CL-0595) and astrocyte cell line C8-D1A (CL-0506) were purchased from KeyGEN BioTECH.

The HT22 cells were cultured in Dulbecco’s modified Eagle’s medium (DMEM; Gibco, Thermo Fisher Scientific, D6429) with high glucose and supplemented with 10% fetal bovine serum (FBS; ExCell Bio, 12B013) and antibiotics (100 U/mL penicillin and 100 µg/mL streptomycin, Gibco) at 37 °C in humidified 5% CO2 incubator. The C8-D1A cells were cultured in Dulbecco’s modified Eagle’s medium (DMEM; Gibco, Thermo Fisher Scientific, D6429) with high glucose and supplemented with 10% fetal bovine serum (FBS; ExCell Bio, 12B013) and antibiotics (100 U/mL penicillin and 100 µg/mL streptomycin, Gibco) at 37 °C in humidified 5% CO2 incubator. Trypsin-EDTA solution (0.25%, Sigma-Aldrich) was used to dissociate HT22 cells and C8-D1A cells. Cells from passage 2-15 were used for the experiments.

C8-D1A cells were stimulated without or with TNFα (PeproTech, 315-01A), IL1β (PeproTech, 211-11B) and FGF1 (Proteintech, 17,400-1-AP) for 24 h and collected for analysis.

For the co-culture experiment [27], one coverslip of FbMSCs (5 × 10^4^ cells), one coverslip of C8-D1A (5 × 10^4^ cells) and one coverslip of neurons (5 × 10^4^ cells) or HT22 (5 × 10^4^ cells) were placed in one 30 mm dish and incubated in the maintenance medium (neural basal medium supplemented with B27 and GlutaMAX). To neutralize the function of FGF1, FGF1 neutralizing antibody (Biotechno, AF-4686) was used in the experiment. FGF1 neutralizing antibody was used at a concentration of 50 ng/mL. Glutamate (AbMole, M10346) were used at a concentration of 100 μM for primary neurons or 20 mM for HT22 cells. Neurons or HT22 cells were collected after two days co-culture and immunofluorescence staining was carried out.

### Evaluation of neurite length and branch count

Random fields of cells were photographed with an Olympus microscope. At least 3 photographs were taken per experimental point and each experiment was repeated at least three times. The number of cells with at least one neurite longer than its cell body was counted. The neurite length of each cell was measured by image J software. Neurites that were longer than a cell nucleus were traced and scaled in micrometers. The average length of neurites was calculated that were measured in each experimental condition.

### Beam walk test

To examine TBI-associated complex motor movements and coordination, a beam walking test was performed as previously described, with modifications [28, 29]. The test was performed at 1–7 days post-TBI. Briefly, the beam was a wooden bar (length: 1200 mm and width: 21 mm) and was placed above the ground. On another end, a black box was placed for animal acclimatization. The mice were allowed to go on the beam to the box and visit it for 60 s. Thereafter, the mice were placed on the beam at a starting distance of 35 cm from the box. The mice were allowed to go to the box and stay there for 60 s. The step was repeated. On the next day, the mice were placed in the box for 60 s and then allowed to go to the box, with the starting point initially at 35 cm, and this was then gradually increased in terms of the distance from the box up to 100 cm. The experiment was repeated three times to cross the beam, with the mice allowed to rest in the box for 1 min. The mean score was calculated from the three runs for each day. The time to cross the beam of the test was counted offline by an observer blinded to the animal treatments.

### Morris water maze test

The Morris water maze (MWM) test was performed as previously described, with modifications [30]. The apparatus used for the MWM test consists of a circular tank filled with water. The water was made opaque by adding a non-toxic white ink. A hidden platform (10 cm in diameter) was placed 1 cm below the water surface during the training in one quadrant of the tank. The mice were trained to memorize the position of the platform; if the animals failed to find the platform, they were guided to the platform and placed on the hidden platform for 30 s. The training session continued for five days and the latency time was calculated. On the 6 th day, the platform was removed, and a probe test was conducted. In the probe trial, the number of crossings, latency to the platform, and time spent in the target quadrant were considered. The data were recorded using a video tracking system (SMART, Panlab Harvard Apparatus, Bioscience Company, Holliston, MA, USA).

### Open field test

In this task, anxiety is reflected by the amount of time rodents spend at the edges of the box, avoiding the center of the open field [31]. Total distance travelled, entries within each zone, and the time spent and the distance traveled within each zone was recorded for 20 min. To test for exploration of a novel chamber, data was analyzed in 5 min bins.

### Novel object recognition test

Fourteen days post-TBI, we used an established protocol to assess each mouse with the NOR test [32, 33]. The experiments were carried out 14 days post-TBI, and the mouse was placed in the box and habituated for 10 min. On the next day, two identical objects (orange cubes, cuboid) were placed in the arena, and the mouse was allowed to explore the area for 10 min. After 1 h, novel object (blue cubes, cylinder) and orange cube were placed in the box, and the mouse was allowed to explore for 5 min while being recorded by camera. We recorded the total time of exploring the novel objects. The time of exploring the novel objects of the test was counted offline by an observer blinded to the animal treatments.

### Immunofluorescence

The immunocytochemistry is performed as described previously. Brain sections were first pretreated in 0.5% Triton X-100 in PBS for 1 h, followed by incubation in 10% normal donkey serum and 0.1% Triton X-100 in PBS for 1 h. Primary antibodies were incubated with brain slices overnight at 4 °C. After additional washing in PBS, the samples were incubated with appropriate secondary antibodies conjugated to Alexa Fluor 488, Alexa Fluor Cy3 for 1 h at room temperature, and then incubated with DAPI (Thermo Fisher, D1306) for 10 min, followed by washing in PBS. The primary antibodies were GFAP (EMD Millipore, 3,380,386, Mouse), MAP2 (Abcam, ab32454, Rabbit), Iba1 (Abcam, ab178847, Rabbit), DCX (Cell Signaling Technology, 91,954, Rabbit) and Active caspase 3 (Bioss, bsm-33199 M, Mouse). The secondary antibodies were Alexa 488 (Jackson ImmunoResearch, 715-546-150) and Cy3 (Jackson ImmunoResearch, 711-165-152).

### Immunofluorescent intensity analysis

Randomly selected high-power fields corresponding to the peri-impact and hippocampus region were used to count positive cells in this region. Briefly, 40 μm cryostat-sectioned tissues were examined at 40 × magnification. Analysis of the mean fluorescence intensity (MFI) for confocal images was conducted using Image J software. From all sections, approximately 4–5 images of were taken from each coronal section using confocal microscopy (Olympus). All photomicrographs were converted to gray scale. Background was selected from blank control images, and subsequently used to subtract the background from all images. All tissue sections were stained within the one batch, with the same imaging threshold and exposure time to ensure maintained consistency for image analysis. Thereafter, the staining intensity of each section was quantified as the average optical density readings of four randomly selected areas within that section. The final staining intensity of each group resulted as the average of each staining intensity per section.

### Quantitative RT-PCR

Cells were collected in Trizol (Invitrogen, 10,296,010) and total RNA was prepared by chloroform extraction and isopropanol precipitation according to the manufacturer’s recommendations [34]. Total RNA converted to cDNAs using a 5 × RT Master Mix (Toyobo, 037,400). Quantitative PCR was performed with 2 × T5 Fast qPCR Mix (SYBR) (TSINGKE, TSE202). Each amplification cycle consisted of an initial step at 95 °C (5 min), followed by 40 cycles of denaturation at 95 °C for 15 s and annealing at 60 °C 1 min, and extension at 72 °C for 30 s. All samples were amplified in duplicate, and every experiment was repeated at least independently 3 times. Relative gene expression was converted using the 2-^△△Ct^ method against the internal control, GAPDH. Primer sequence list was shown in Additional file 2: Table S1 [see Additional file 1].

### Cell viability staining

We used the live-dead cell imaging kit (Thermo Fisher, L3224) to evaluate cell viability [35].

### Glutamate assay

Glutamate assay kit (Invitrogen, MAK004-1KT) from Sigma was used to quantify glutamate concentration in C8 cells according to the manufacturer’s instruction [36, 37].

### Western blot

Mice were scarified 4 days after TBI (*n* = 3/group). The brains were removed, and the lesion areas were isolated for protein preparation. Western blotting was carried out as previously described [38]. Brain tissue were lysed using RIPA Lysis Buffer with CompleteTM EDTA-free protease inhibitor (Roche, 05,892,791,001). The protein concentration was determined using a BCA protein assay kit (Thermo Fisher, 23,225). Whole-proteome fractions from samples were subsequently separated by SDS-PAGE using 12% Tris gels, blotted onto PVDF membranes and incubated with the appropriate antibodies overnight. After incubation with relevant secondary antibodies, the labeled proteins were visualized using an ECL chemiluminescence detection kit (Merck Millipore, 34,075). The following antibodies were used: anti-GAPDH (ABclonal, A19056, Rabbit, 1:1000), anti-BDNF (Proteintech, 28,205-1-AP, Rabbit, 1:1000), anti-FGF1 (Proteintech, 17,400-1-AP, Rabbit, 1:400).

### Transfections and treatments of FbMSCs

Cells were seeded in 6-well plates 24 h before transfection[39]. The cells were transfected with 100 nM commercially synthesized FGF1-siRNA (siFGF1) or negative control (siNC) (HANBIO, China) using RNAFit (HB-RF-1000) in serum-free Opti-MEM. The Opti-MEM medium was replaced with FBS-containing medium after incubation for 6 h. FbMSCs were cultured for another 48 h after the transfection, and the knockdown efficiency was examined by qRT-PCR and Western blot. The sequences are as follows: negative control forward, 5’-UUCUCCGAACGUGUCACGUTT-3’ and reverse, 5’-ACGUGACACGUUCGGAGAATT-3’; FGF1-siRNA forward, 5’-CGGGCGAAGUGUAUAUAAATT-3’ and reverse, 5’- UUUAUAUACACUUCGCCCGTT-3’.

### Statistical analysis

Data are presented as mean ± SEM. Two-way ANOVA was used to analyze all behavioral tests between and among the treatment groups. In anatomical and biochemical studies, one-way or two-way ANOVA was used to compare multiple groups. A Bonferroni post hoc analysis was used to determine whether differences were significant. Differences between two groups were tested with the two-tailed Student’s t-test. The criteria for statistical significance were *p* < 0.05. *, **, ***, and **** indicate significance at *p* < 0.05, *p* < 0.01, *p* < 0.001, and *p* < 0.0001, respectively.

## Results

### Characteristics of ectoderm-derived FbMSCs

**Fig. 1:**
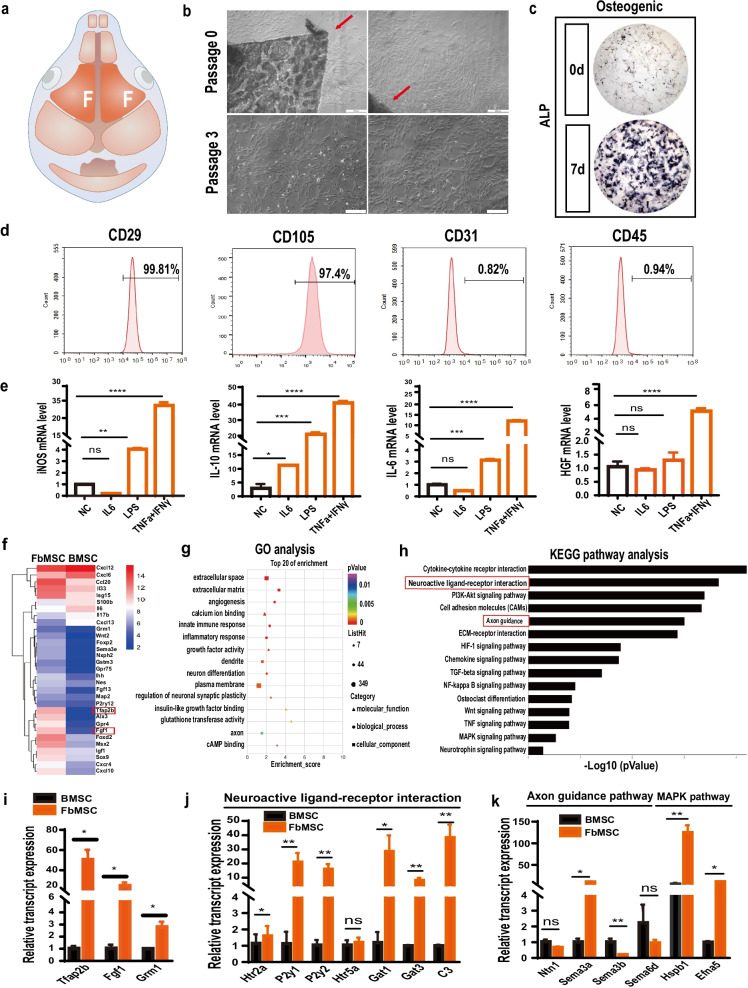
Characteristics of frontal bone mesenchymal stem cells (FbMSCs). **a** Schematic of murine skull. Areas marked F refer to frontal bone, from where FbMSCs were isolated. **b** Representative morphological features of FbMSCs. Scale bar, 200 μm. **c** ALP staining of FbMSCs. Scale bar, 50 μm. **d** Flow cytometric analysis of FbMSCs. **e** Expression of iNOS, IL-10, IL6, and HGF in FbMSCs stimulated with the indicated factors. Microarray analysis was performed on FbMSCs and bone marrow MSCs (BMSCs). **f** Cluster heat map of representative differential genes in FbMSCs and BMSCs. GO (**g**) and KEGG (**h**) analysis of enriched pathway in FbMSCs. Quantitative RT-PCR verified the higher expression of Tfap2β, Fgf1, and Grm1 (**i**), as well as genes related to the neuron ligand-receptor pathway (**j**), axon guidance pathway and MAPK pathway (**k**) in FbMSCs. (Data are presented as the mean ± standard error; *, **, ***, and **** indicate significance at *p* < 0.05, *p* < 0.01, *p* < 0.001, and *p* < 0.0001, respectively).

Ectoderm-derived FbMSCs isolated from the frontal bone of neonatal mice exhibited fibroblast-like morphology (Fig. 1a, b). After cultured in osteoblast induction conditions, FbMSCs were positive for ALP staining (Fig. 1c). Flow cytometric analysis showed that FbMSCs were positive for mesenchymal makers CD29 and CD105, but negative for hematopoietic and endothelial cell lineage markers CD31 and CD45, respectively (Fig. 1d). To investigate the potential immunoregulatory function, FbMSCs were stimulated with different factors. As shown in Fig. 1e, the expression of iNOS, IL-10, IL6, and HGF was significantly increased with TNFα and IFNγ stimulation. Transcriptomic analysis using an Affymetrix Clariom D array was performed to examine the transcripts in FbMSCs and bone marrow MSCs (BMSCs). Heat map assays identified the differences of gene expression between FbMSCs and BMSCs. The ectodermal-specific transcription factor Tfap2β and growth factor FGF1 were highly expressed in FbMSCs (Fig. 1f), which was confirmed by qRT-PCR analysis (Fig. 1i). GO and KEGG enrichment analysis demonstrated differences in nervous system-related pathways such as neuroactive ligand-receptor interaction and axon guidance (Fig. 1g, h), which were confirmed by qRT-PCR analysis (Fig. 1j, k). Microarray analysis was also performed on FbMSCs and parietal bone derived MSCs (PbMSCs), which were mesoderm-derived. Compared to PbMSCs, FbMSCs also showed a higher expression of Tfap2β and FGF1 (Additional file 3: Fig. S1).

### FbMSC application improves learning and memory deficits in TBI mice

**Fig. 2:**
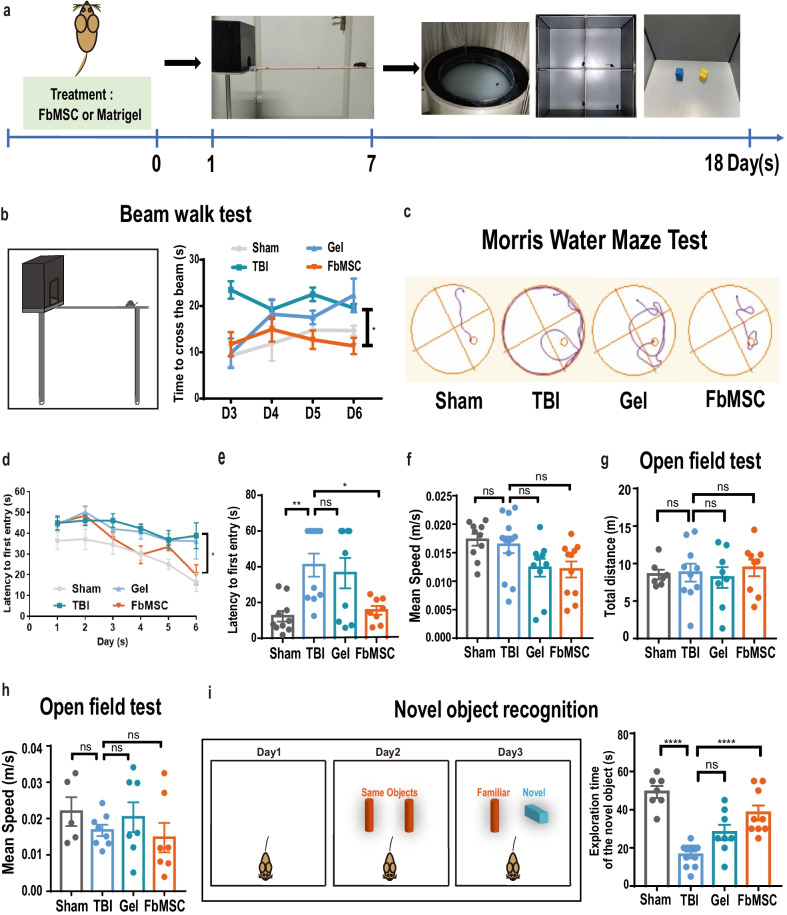
FbMSC transplantation improved learning and cognitive ability in TBI mice. **a** Experimental schedule. FbMSCs were transplanted immediately after TBI (D0). All mice underwent behavioral tests at the indicated days. **b** Schematic of beam walk test (left) and latency to cross the beam (right). **c**–**f** Morris water maze test was used to evaluate the learning and cognitive ability of mice. Typical escape route map (**c**), learning curve (**d**), escape latency (**e**), and movement speed (**f**) of each group were displayed. Total distance (**g**) and mean speed (**h**) of each group in the open field test. **i** Schematic (left) and results (right) of novel object recognition experiment. (*n* = 8–10 mice per group; Data are presented as the mean ± standard error; *, **, ***, and **** indicate significance at *p* < 0.05, *p* < 0.01, *p* < 0.001, and *p* < 0.0001, respectively).

We explored the therapeutic effects of FbMSCs in the cortical impact injury (CCI) TBI mouse model. CCI was performed on adult C57BL/6 N mice in a moderate TBI model. After brain injury, mice showed deficient learning and memory abilities (Additional file 4: Fig. S2), increased Iba1 and GFAP immunofluorescence staining intensity, and decreased microtubule associated protein 2 (MAP2) staining (Additional file 5: Fig. S3). FbMSCs mixed with Matrigel or Matrigel alone were transplanted to the injury site immediately after the injury. Various behavioral experiments, including beam walk test (BWT), Morris water maze test (MWMT), open field test (OFT), and novel object recognition (NOR) experiment, were conducted to examine the spatial learning and memory abilities of all the four TBI groups (Sham group, TBI alone, Matrigel-treated TBI, and FbMSC-treated TBI; Fig. 2a). The movement and coordination abilities of all groups were evaluated using the beam walk test, which revealed that the FbMSC-treated TBI group had an improved motor coordination ability (Fig. 2b). Typical escape routes in the MWM test were shown in Fig. 2c. The escape latency of the FbMSC-treated TBI group was similar to that of the Sham group but much faster than that of the TBI group and Matrigel-treated TBI group (Fig. 2d, e). However, no significant differences were identified in movement speed among the four groups (Fig. 2f). In the open field test, the total distance and the movement speed in the field were similar among the four groups (Fig. 2g, h). The novel object recognition test is a learning and memory test based on the principle that animals have a natural tendency to explore novel object [40]. The time of the FbMSC-treated TBI group to explore novel objects was significantly improved, compared with that of the TBI group (Fig. 2i). These results suggested that FbMSC transplantation could improve the learning and cognitive function of TBI mice, as well as their motor coordination ability to a certain extent.

### Transcriptional profile after FbMSC transplantation

**Fig. 3:**
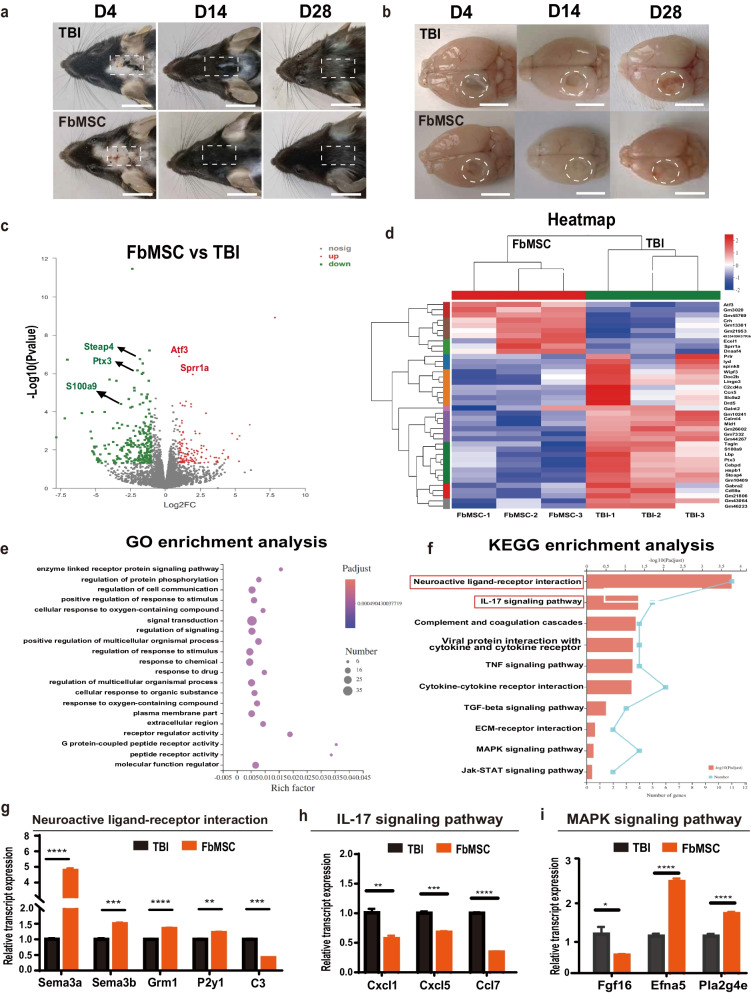
RNA sequencing analysis of FbMSC-treated TBI group and TBI group. Representative photos of wound healing (**a**) and topical views of brains (**b**) from the indicated groups collected at days 4, 14, and 28 after TBI. Scale bar, 2 mm. Volcano maps (**c**) and heat map (**d**) of differentially expressed genes in FbMSC-treated TBI group and TBI group. **e** GO analysis showed enrichment in cellular component, molecular function and biological process. **f** KEGG analysis of enriched pathway in FbMSCs. Quantitative RT-PCR verified the expression genes related to neuroactive ligand-receptor interaction (**g**), IL-17 signaling pathway (**h**) and MAPK signaling pathway (**i**). (*n* = 3–4 mice per group; Data are presented as the mean ± standard error; *, **, ***, and **** indicate significance at *p* < 0.05, *p* < 0.01, *p* < 0.001, and *p* < 0.0001, respectively).

Corresponding to the improved behavior, the wound size and area were both significantly reduced to healthy levels with FbMSC transplantation, whereas the TBI group only showed partial recovery (Fig. 3a, b). Brain tissues around the injured area from FbMSC-treated group and TBI group were collected and processed for subsequent downstream bulk RNA sequencing (RNA-seq). Overall, many differentially expressed genes were found when the transcriptomes of the both groups were compared. Volcano plot (Fig. 3c) depicted the significantly upregulated and downregulated mRNAs, some significantly up-regulated genes and down-regulated genes were marked. The Heatmap revealed the differences in the relative gene expression between FbMSC-treated TBI and TBI group (Fig. 3d). GO and KEGG enrichment analysis revealed significant differences in neuroactive ligand-receptor interaction and IL-17 signaling pathway between FbMSC-treated TBI and TBI group (Fig. 3e, f). Genes related to the relative pathways were confirmed by qRT-PCR analysis (Fig. 3g–i).

### FbMSC transplantation enhances neurogenesis and synaptic remodeling

**Fig. 4:**
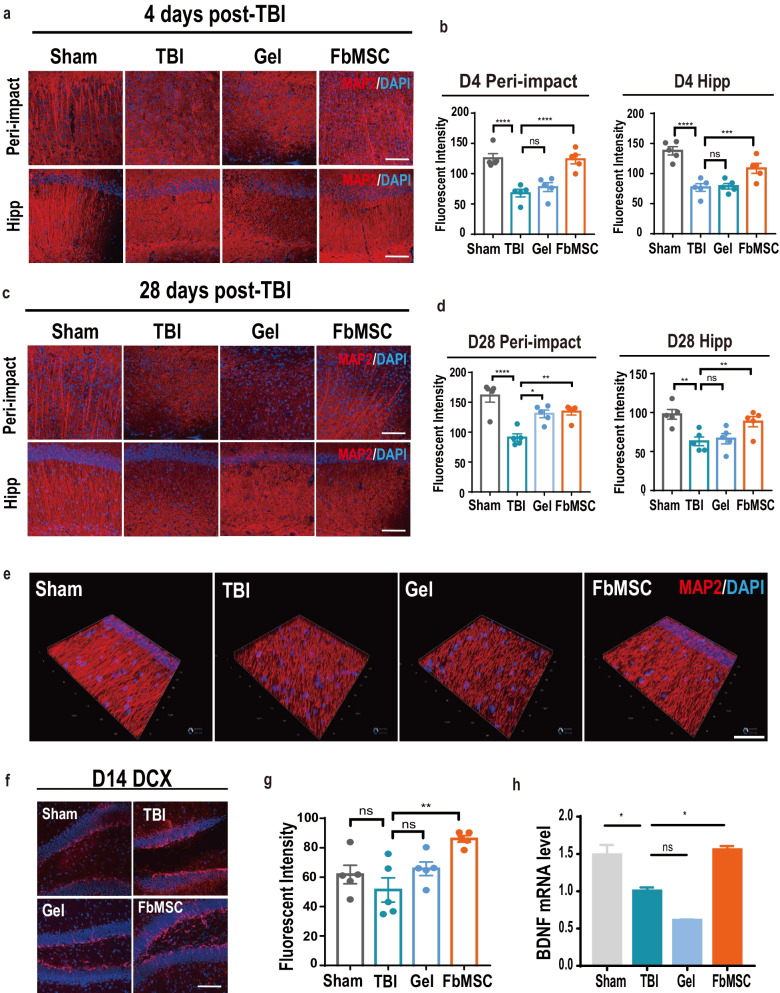
FbMSC application promotes the recovery of axonal injury in TBI mice. MAP2 immunofluorescence and fluorescence intensity quantification in peri-impact area and hippocampus in each group at 4 d (**a, b**) and 28 d (**c, d**) post-injury. Scale bar, 20 μm. **e** Representative 3D reconstruction images of MAP2 staining in hippocampus in each group at 4 d post-injury. Scale bar, 150 μm. Doublecortin (DCX) immunofluorescence image (**f**) and fluorescence intensity quantification (**g**) in each group at 14 d post-injury. Scale bar, 20 μm. **h** Expression of BDNF was detected by qRT-PCR at 14 d post-injury. (*n* = 3–4 mice per group; Data are presented as the mean ± standard error; *, **, ***, and **** indicate significance at *p* < 0.05, *p* < 0.01, *p* < 0.001, and *p* < 0.0001, respectively.)

Neuron death and diffuse axonal injury are common complications of TBI [41]. To quantify mature neurons, MAP2 staining was performed at 4 d and 28 d post-injury in the injured area and hippocampus. The labeling intensity of MAP2 in the injured area and hippocampus was significantly decreased in the TBI group at 4 d (Fig. 4a, b) and 28 d (Fig. 4c, d) post-injury. The TBI group had significantly lower MAP2 staining intensities than the Sham group in both the injured area and hippocampus. MAP2 staining quantification of the FbMSC-treated group were comparable to those in the Sham group, which indicated that FbMSC transplantation dramatically promoted neuron recovery. 3D construction of MAP2 staining confirmed the recovery of neurons with FbMSC application (Fig. 4e). Newly generated neurons from hippocampal neurogenesis are thought to play a crucial role in some types of learning and memory [42, 43]. We used DCX to label the developing and migrating neurons. The labeling intensity of DCX in the FbMSC-treated TBI group was more robust than that in the TBI group (Fig. 4f, g). In addition, the level of BDNF at 14 d post-injury was higher in the FbMSC-treated TBI group than in the TBI group (Fig. 4h). These results revealed that FbMSC treatment could promote neurogenesis and synaptic remodeling.

### FbMSC transplantation reduces the activation of microglia

**Fig. 5:**
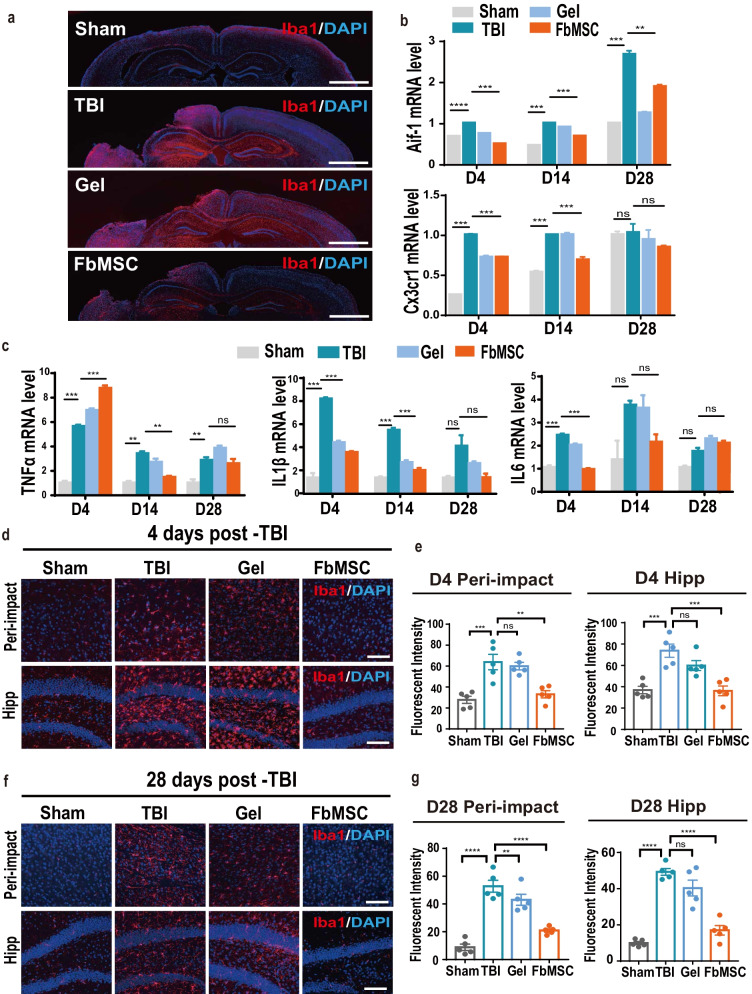
FbMSC application decreased the activation of microglia in TBI mice. **a** Representative Iba1-stained coronal sections of murine brains of the four groups at 4 d post-injury. Scale bar, 2 mm. Expression of Aif1, Cx3cr1 (**b**) and pro-inflammatory factors TNFα, IL1β, IL6 (**c**) was detected by qRT-PCR at 4 d, 14 d, and 28 d post-injury. Iba1 immunofluorescence and mean fluorescence intensity quantification in peri-impact area and hippocampus in each group at 4 d (**d, e**) and 28 d (**f, g**) post-injury. Scale bar, 20 μm. (*n* = 3–4 mice per group; Data are presented as the mean ± standard error; *, **, ***, and **** indicate significance at *p* < 0.05, *p* < 0.01, *p* < 0.001, and *p* < 0.0001, respectively.)

Severe neuroinflammation exacerbates brain injury. Microglia are resident immune cells in the brain that play an important role in regulating neuroinflammation or injury. Iba1 labeling was used to evaluate microglia activation in the injured brain (Fig. 5a). Aif-1 and Cx3cr1 expression levels in the injured tissues at 4 d, 14 d, and 28 d after TBI were quantified to further confirm microglial activation. As shown in Fig. 5b, the expression levels of Aif-1 and Cx3cr1 were increased in the TBI group but significantly decreased in the FbMSC-treated TBI group, compared to those in the Sham group. We also examined the expression of pro-inflammatory factors in brain tissue post-injury. Overall, the levels of TNFα, IL1β, and IL6 in the FbMSC-treated TBI group were significantly decreased compared to those in the TBI group (Fig. 5c). Activated microglia were Iba1-positive and showed enlarged cell bodies and axon coarseness. The labeling intensity of Iba1 in the injured area and hippocampus was significantly increased in the TBI group at 4 d (Fig. 5d, e) and 28 d (Fig. 5f, g) post-injury. Immunofluorescence staining and the intensity quantification data showed that FbMSC transplantation dramatically reduced microglial activation. These results indicated that FbMSCs could restrict the activation of microglia to a certain extent.

### FbMSC application alleviates the activation of astrocytes

**Fig. 6:**
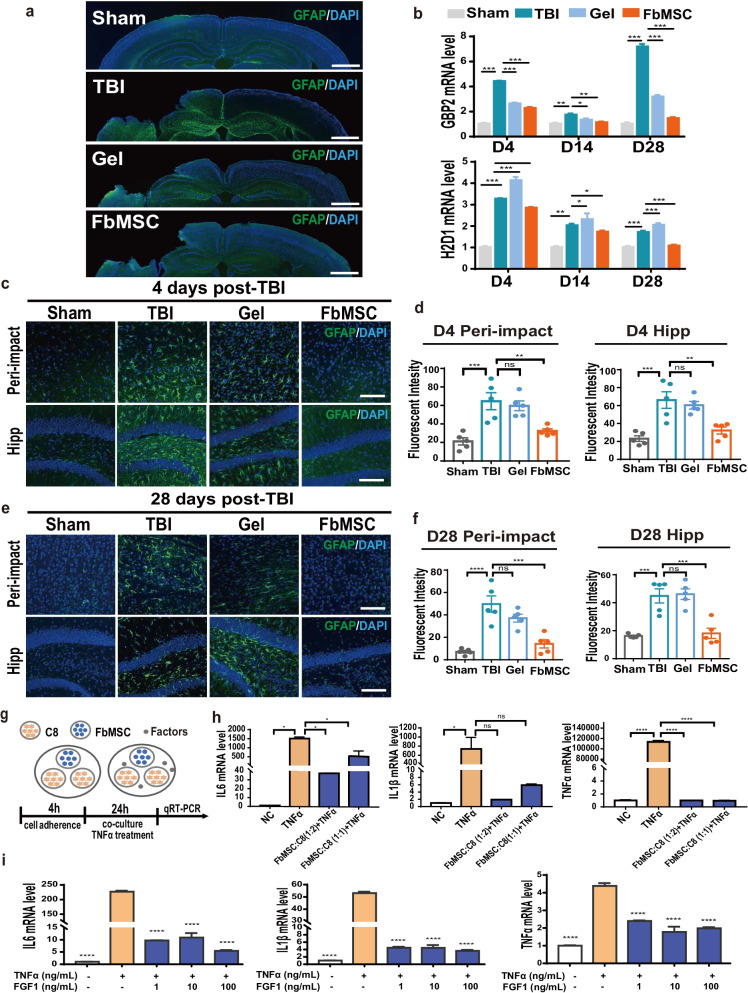
Application of FbMSCs decreased the activation of astrocytes. **a** Representative GFAP stained coronal sections of murine brains of the four groups at 4 d post-injury. Scale bar, 2 mm. **b** Expression of H2D1 and GBP2 was detected by qRT-PCR at 4 d, 14 d, and 28 d post-injury. GFAP immunofluorescence and fluorescence intensity quantification in peri-impact area and hippocampus in each group at 4 d (**c, d**) and 28 d (**e, f**) post-injury. Scale bar, 20 μm. **g** Schematic of FbMSCs and astrocyte cell line C8-D1A co-culture. **h** C8-D1A cells were collected after 24 h co-culture. IL6, IL1β, and TNFα expression was detected by qRT-PCR. **i** IL6, IL1β and TNFα expression was detected after TNFα (150 ng/mL), IL1β (50 ng/mL) and FGF1 (1, 10, 100 ng/mL) induction. (*n* = 3–4 mice per group; Data are presented as the mean ± standard error; *, **, ***, and **** indicate significance at *p* < 0.05, *p* < 0.01, *p* < 0.001, and *p* < 0.0001, respectively).

Brain injury is generally accompanied by reactive astrogliosis [44, 45]. We used glial fibrillary acidic protein (GFAP) to label astrocytes in the brain tissues of the four groups (Fig. 6a). The levels of A1 astrocyte-specific transcripts H2D1, and GBP2 were significantly decreased in the FbMSC-treated TBI group (Fig. 6b). As revealed by GFAP immunofluorescence, the appearance of astrocytes with increased sizes and enlarged branches was mostly observed in 4 d (Fig. 6c) and 28 d (Fig. 6e) in TBI and Matrigel-treated TBI groups. GFAP labeling intensity in the FbMSC-treated TBI group was almost similar to that in the Sham group but significantly lower than that in the TBI and Matrigel-treated TBI groups (Fig. 6d, f). Brain tissues around the injury site were collected to examine the activation of astrocytes using qRT-PCR. Different doses of FbMSCs were co-cultured with the astrocyte cell line C8-D1A and stimulated with TNFα (150 ng/mL) and IL1β (50 ng/mL) (Fig. 6g). The results revealed that FbMSCs could significantly suppress the expression of proinflammatory factors IL6, IL1β, and TNFα in C8-D1A cells (Fig. 6h). In addition, the levels of IL6, IL1β, and TNFα were decreased after FGF1 induction (1, 10 and 100 ng/mL) (Fig. 6i). These results indicated that FbMSCs could restrict the activation of astrocyte to a certain extent.

### FGF1 secreted from FbMSCs alleviates glutamate excitotoxicity to neurons by promoting glutamate transportation in astrocytes

**Fig. 7:**
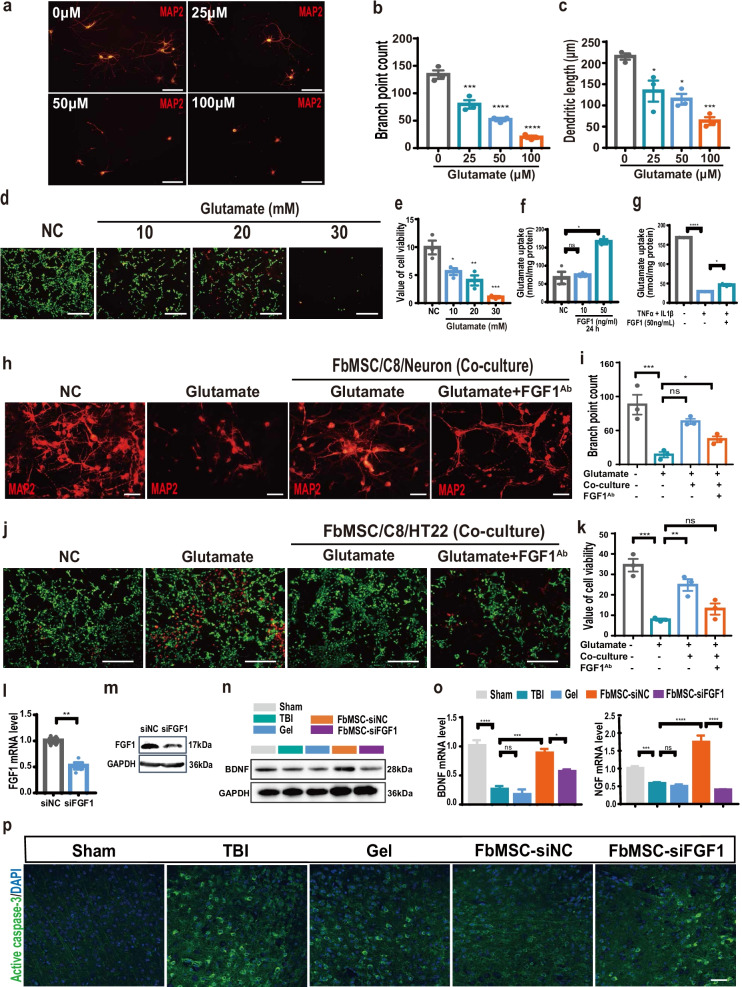
FGF1 from FbMSCs promotes glutamate uptake of C8-D1A cells and alleviates neuron excitotoxicity. **a** Representative images of MAP2 stained neurons treated with indicated doses of glutamate. Scale bar, 20 μm. Branch point (**b**) and Dendritic length (**c**) were quantified according to MAP2 staining. Representative staining images (**d**) and quantification of cell viability (**e**) according to calcein-AM (green)/ethidium homodimer (red) staining on HT22 cells. Scale bar, 100 μm. **f** Glutamate uptake of C8-D1A cells stimulated with indicated doses of FGF1. **g** Glutamate uptake of C8-D1A cells stimulated with indicated dose of TNFα (150 ng/mL), IL1β (50 ng/mL) and FGF1 (50 ng/mL). Representative images (**h**) and quantification of neuron branches (**i**) of MAP2-stained neurons in indicated groups. Scale bar, 20 μm. Representative images (**j**) and quantification of cell viability (**k**) of calcein-AM (green)/ethidium homodimer (red) staining of HT22 cells in the indicated groups. Scale bar, 100 μm. **l** Expression level of FGF1 mRNA in FbMSCs after transfected with siFGF1 and siNC. **m** Western blot analysis demonstrated levels of FGF1 after transfected with siFGF1 and siNC. **n** Western blot analysis demonstrated levels of BDNF in lesion areas of mice at 4 days. **o** mRNA level of BDNF and NGF was detected by qRT-PCR. **p** Representative images of active caspase-3 staining in the lesion areas from each group at 4 days. Scale bar, 20 μm. (*n* = 3–4 mice per group; Data are presented as the mean ± standard error; *, **, ***, and **** indicate significance at *p* < 0.05, *p* < 0.01, *p* < 0.001, and *p* < 0.0001, respectively.)

In addition to increased neuroinflammation, the excessive release of glutamate is also responsible for the secondary neuronal death in TBI [46]. To verify the effect of glutamate on neurons, primary neurons were treated with different doses of glutamate and then stained with MAP2. The number of cytoskeleton branches of the neurons was significantly decreased with glutamate treatment in a dose-dependent manner, with more glutamate resulting in fewer branches (Fig. 7a–c). We then treated the neuron cell line HT22 with glutamate and performed live and dead assay via calcein-AM (green, live)/ethidium homodimer (red, dead) staining. As shown in Fig. 7d, e, more red staining appeared in cells treated with glutamate, indicating the cytotoxicity of glutamate on HT22 cells. Previous studies [47] and our data demonstrated that FGF1 exposure promoted glutamate transport by astrocyte cell line C8-D1A (Fig. 7f, g). Compared with MSCs from the bone marrow and parietal bone, FbMSCs highly expressed FGF1 (Fig. 1f, i, Additional file 3: Fig. S1). To evaluate the influence of FGF1 from FbMSCs on astrocytes, we co-cultured FbMSCs, astrocyte cell line C8-D1A, and primary neurons in the presence of glutamate for 24 h. MAP2 staining data and neuron branches counting revealed that the presence of FbMSCs and C8-D1A co-cultured reduced the cytotoxicity of glutamate on neurons (Fig. 7h, i). To explore whether FGF1 secreted from FbMSCs mediated the recovery of neurological damage, a neutralizing antibody against FGF1 (FGF1^Ab^) (5 μg/mL) was added to the FbMSCs, C8-D1A, and neuron co-culture system. The protective effect of FbMSCs and C8-D1A was compromised with FGF1^Ab^ addition (Fig. 7h, i). We also co-cultured FbMSCs, astrocyte cell line C8-D1A, and HT22 cells in the presence of glutamate for 24 h and performed live/dead staining. Similarly, FbMSCs and C8-D1A co-culture alleviated the cytotoxicity of glutamate on HT22 cells. The presence of FGF1^Ab^ reduced the protective effect of FbMSCs and C8-D1A (Fig. 7j, k). To further confirm the function of FGF1, FbMSCs was transfected with siRNA for reducing FGF1 expression and then transplanted for TBI therapy. As shown in Fig. 7l, m, compared to the counterpart, the mRNA and protein levels of FGF1 in FbMSCs were significantly reduced after transfected with FGF1-siRNA. Western blot data showed that BDNF protein level in FbMSC-siFGF1 treated TBI group was lower than that in FbMSC-siNC treated TBI group (Fig. 7n). The mRNA levels of BDNF and NGF were also significantly lower in FbMSC-siFGF1 group than in FbMSC-siNC group (Fig. 7o). In addition, TBI induced neuronal apoptosis which could attenuated by FbMSC-siNC instead of by FbMSC-siFGF1 treatment (Fig. 7p). Collectively, these data revealed an important role of FGF1 from FbMSCs in mediating the protective effect on neurons.

## Discussion

**Fig. 8:**
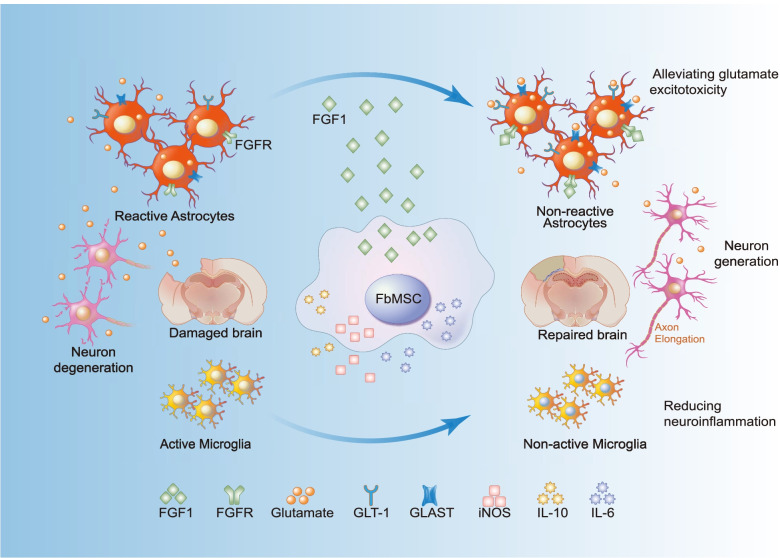
Proposed mechanism. FbMSC application reduces the activation of microglia cells and astrocytes and alleviates glutamate excitotoxicity. Abbreviations: FGF1, acidic fibroblast growth factor1; FGFR, acidic fibroblast growth factor1 receptor; GLT-1, glutamate transporter-1; GLAST, glutamate-aspartate transporter; iNOS, inducible nitric oxide synthase; IL6, interleukin 6; IL-10, interleukin-10

In this study, we found that ectoderm-derived FbMSC application in TBI mice significantly inhibited neuroinflammation, promoted neurogenesis, improved cognition and behavioral deficits. Additionally, FGF1 from FbMSCs enhanced glutamate transportation in astrocytes, contributing to the alleviation of glutamate cytotoxicity in neurons (Fig. 8).

TBI pathogenesis includes a complex process from primary to secondary injuries, leading to anatomical and functional brain damage [48]. Numerous therapeutic strategies for alleviating inflammation, scavenging reactive oxygen species, and promoting tissue regeneration have been developed for TBI recovery [49]. Of these, stem cell-based therapy provided an ideal therapeutic option for neurological disorders based on its ability to inhibit neuroinflammation and promote tissue reconstruction. In the present study, we found that FbMSCs isolated from the frontal bone demonstrated some characteristics of NSCs, and were ectoderm-derived and could differentiate into different types of neurons (data not shown). FbMSCs also showed properties similar to those of MSCs, including the secretion of anti-inflammatory factors under TNFα and IL1β stimulation and differentiation into osteoblasts. Our data showed that in situ FbMSC application suppressed microglia and astrocyte activation, promoted neural regeneration, and alleviated behavioral deficits in TBI mice.

Immediately following TBI, both the peripheral immune system and the neuroimmune system react to create an inflammatory response to promote mobilization of additional immune cells and to clear cellular debris. Controlling the immune response will help to stop the prolonged and exacerbated pathological response to injury. MSCs display a wide range of immune-modulatory capabilities [50]. Similar to MSCs from mesoderm, FbMSC transplantation inhibited the activation of both microglia cells and astrocytes showing as decreased expression of inflammatory factors, less intensity of both Iba1 and GFAP staining until 28 days post TBI. After FbMSC treatment, the expression of H2D1 and GBP2 in brain tissue and the level of IL6, IL1β, and TNFα in astrocytes decreased significantly, which indicated the reduced polarization of astrocytes to A1 like phenotypes. Several previous studies have demonstrated the effectiveness of using MSCs to treat TBI by characterizing the cell source, delivery route, dosage, and timing of delivery. Ruppert et al. explored the immunomodulatory potential of early and delayed MSC administration as a treatment for TBI. Their data indicated that both the early and delayed MSC infusion groups benefitted significantly [51]. Hopefully, the delayed FbMSC transplantation for TBI will also improve the injury for the immunosuppressive function of FbMSCs.

Mesoderm-derived MSCs have been extensively investigated in TBI models owing to their abundance, multiple differentiation abilities, immunoregulatory capacities, and easily available from various tissues (i.e., bone marrow, umbilical cord, and adipose tissue) [11, 52, 53]. Previous studies have demonstrated that stem cells from the same lineage are more effective for post-injury recovery [18, 19]. Leucht et al. reported that neural crest stem cells contributed exclusively to the regeneration of neural crest-derived skeletal elements, indicating that reparative strategies should focus on lineage origin to maximize the effectiveness of recovery treatment [18]. NSCs and their derived-neurons, oligodendrocytes and astrocytes originate from the ectoderm. Here, we isolated and investigated the reparative function of ectoderm-derived FbMSCs in TBI mice. RNA-seq and qRT-PCR data showed that FbMSCs highly expressed the ectodermal cell-specific transcription factor Tfap2β, which is not expressed in mesoderm-derived cells. Similar to those of bone marrow MSCs, after induction by TNFα and IL1β, FbMSCs produced iNOS and IL-10, which have been reported to play important roles in suppressing excessive immune responses [54, 55].

In the central nervous system, excessive glutamate release is responsible for the secondary neuronal death following neuronal injury. Glutamate excitotoxicity in the acute phase of TBI generally aggravates neuronal loss. Different strategies have been explored to protect neurons from glutamate excitotoxicity. VEGF from NSCs contributes to stroke recovery by promoting the upregulation of GLT-1 expression in astrocytes and reducing peri-ischemic extracellular levels of glutamate [56, 57]. In the present study, we found that FGF1 secreted from FbMSCs could enhance glutamate transportation by astrocyte cell line C8-D1A. RNA-seq assays revealed that FbMSCs expressed higher levels of FGF1 than bone marrow MSCs. In vitro analysis showed that FGF1 promoted GLT-1 expression in astrocytes and enhanced glutamate transportation. Previous studies have shown that FGF1 was found in preadipocytes [58], astrocytes [47], and neural crest-derived osteogenic progenitors [18] and could minimize the number of apoptotic cells after injury. Our results revealed that addition of FGF1 antibody attenuated the protective effect of C8-D1A and FbMSCs.

Nevertheless, there are some limitations in the current study. First, delivery of FbMSCs right after TBI is a translational limitation. It will be helpful to further investigate the effect of delayed application of FbMSCs for TBI therapy. Second, cell lines do not replicate all the key physiological behaviors of primary cells. Although both primary astrocytes and C8-D1A can uptake glutamate, studies between primary astrocytes and FbMSCs will further strengthen the function of FbMSCs.

## Conclusions

In summary, this is the first report on applying ectoderm-derived FbMSCs in a TBI model to alleviate anatomical and functional injury. FbMSCs effectively ameliorated structural and functional injury, markedly reduced neuroinflammation, alleviated excessive glutamate excitotoxicity, and mitigated neuronal damage. Additionally, FGF1 from FbMSCs promoted the transportation of glutamate by astrocytes, mitigating the continuous neuronal loss. Overall, our findings highlight the therapeutic potential of ectoderm-derived FbMSCs for TBI which mostly affect cells from the same embryonic origins as FbMSCs.

## Supplementary Information

**Additional file 1:** Supplemental Figure and Table legends.

**Additional file 2:** Sequences of primers used for qRT-PCR analysis of mRNA levels.

**Additional file 3:** Affymetrix Clariom D array showed differences between FbMSCs and PbMSCs.

**Additional file 4:** Traumatic brain injury damages the learning and cognitive ability of mice.

**Additional file 5:** Adverse changes of brain microenvironment in mice with brain injury.

## Data Availability

The datasets used and/or analyzed during the current study are available from the corresponding author upon reasonable request.

## Abbreviations

GFAP: Glial fibrillary acidic protein
LPS: Lipopolysaccharide
DAPI: 4′,6-Diamidino-2-phenylindole
Iba1: Ionized calcium-binding adaptor molecule 1
MWMT: Morris water maze test
TBI: Traumatic brain injury
FbMSCs: Frontal bone mesenchymal stem cells
CCI: Controlled cortical impact
NSC: Neural stem cell
NPC: Neural progenitor cell
MSC: Mesenchymal stem cell
iNOS: Inductible nitric oxide synthase
IL-10: Interleukin-10
IL6: Interleukin 6
IL1β: Interleukin 1β
TNFα: Tumor necrosis factor alpha
IFN-γ: Interferon-gamma
HGF: Hepatocyte growth factor
PbMSCs: Parietal bone derived MSC
MAP2: Microtubule associated protein 2
DCX: Doublecortin X
BDNF: Brain-derived neurotrophic factor
NGF: Nerve growth factor
Cx3cr1: C-X3-C motif chemokine receptor 1
Aif-1: Allograft inflammatory factor-1
FGF1: Fibroblast growth factor 1
Tfap2β: Transcription factor activating enhancer-binding protein 2β
VEGF: Vascular endothelial-derived growth factor
GLT-1: Glutamate transporter 1
PEG2: Prostaglandin E2
NO: Nitric oxide
