# Supplementary material for: Ectoderm-derived frontal bone mesenchymal stem cells promote traumatic brain injury recovery by alleviating neuroinflammation and glutamate excitotoxicity partially via FGF1
Source: Stem Cell Res Ther. 2022 Jul 26;13:341. doi: 10.1186/s13287-022-03032-6 (PMC9327213; doi:10.1186/s13287-022-03032-6)
Supplement: Supplementary file 1 — Additional file 1: Supplemental Figure and Table legends. [file 13287_2022_3032_MOESM1_ESM.docx]

**Ectoderm-derived frontal bone mesenchymal stem cells promote traumatic brain injury recovery by alleviating neuroinflammation and glutamate excitotoxicity partially via FGF1**

Qiaozhen Qin^1,2†^, Ting Wang^1†^, Zhenhua Xu^1†^, Shuirong Liu^1†^, Heyang Zhang^1^, Zhangzhen Du^1^, Jianing Wang^1^, Yadi Wang^1^，Zhenning Wang^1^, Shanshan Yuan^1^, Jiamei Wu^1^, Wenyan He^5^, Changzhen Wang^4^, Xinlong Yan^2*^, Yan Wang^1,3*^, Xiaoxia Jiang^1,3*^

1. Beijing Institute of Basic Medical Sciences, 27 Taiping Road, Haidian District, Beijing 100850, P.R. China.

2. Faculty of Environmental and Life Sciences, Beijing University of Technology, Beijing 100124, China.

3. Anhui Medical University, Hefei 230032, Anhui, China.

4. Beijing Institute of Radiation Medicine, 27 Taiping Road, Haidian District, Beijing 100850, P.R. China.

5. China National Clinical Research Center for Neurological Diseases, Jing-Jin Center for Neuroinflammation, Beijing Tiantan Hospital, Capital Medical University, Beijing, China.

† These authors contribute equally to this work.

**Corresponding author:**

Xiaoxia Jiang Email: [smilovjiang@163.com](mailto:smilovjiang@163.com) Yan Wang Email: yan_way@126.com

Xinlong Yan Email: [yxlong2000@bjut.edu.cn](mailto:yxlong2000@bjut.edu.cn)

**Supplemental Figures and Tables**

**Figure S1. Affymetrix Clariom D array showed differences between FbMSCs and PbMSCs.**

(**a**) Cluster heat map of representative differential genes in FbMSCs and PbMSCs. GO (**b**) and KEGG (**c**) analysis of enriched pathway in FbMSCs.

**Figure S2. Traumatic brain injury damages the learning and cognitive ability of mice.**

(**a**) Experimental schedule. All mice underwent behavioral tests at the indicated days. (**b-e)** Morris water maze test was used to evaluate the learning and cognitive ability of mice. Typical escape route map (**b**), escape latency (**c**), learning curve (**d**), and movement speed (**e**) were displayed. (**f**) Time in the center zone (left), average speed (middle), and total distance (right) of each group in the open field test. (n = 4-10 mice per group; Data are presented as the mean ± standard error; *, **, ***, and **** indicate significance at p < 0.05, p < 0.01, p <0.001, and p <0.0001, respectively.)

**Figure S3. Adverse changes of brain microenvironment in mice with brain injury.**

(**a**) Real time quantitative PCR (qRT-PCR) was used to detect IL6 (left) and IL1β (middle), TNFα (right). Iba1 (**b**), GFAP (**c**), and MAP2 (**d**) immunofluorescence and fluorescence intensity quantification in peri-impact area in each group at 1, 3, 5 d post-injury. Scale bar, 20 μm. (n = 3-4 mice per group; Data are presented as mean ± standard error; *, **, ***, and ****indicate significance at p < 0.05, p < 0.01, p < 0.001, and p < 0.0001, respectively.)

**Table S1. Sequences of primers used for qRT‐PCR analysis of mRNA levels**

Table S1 lists the primer sequences used in the experiment.
