# Supplementary material for: Ectoderm-derived frontal bone mesenchymal stem cells promote traumatic brain injury recovery by alleviating neuroinflammation and glutamate excitotoxicity partially via FGF1
Source: Stem Cell Res Ther. 2022 Jul 26;13:341. doi: 10.1186/s13287-022-03032-6 (PMC9327213; doi:10.1186/s13287-022-03032-6)
Supplement: Supplementary file 2 — Additional file 2: Sequences of primers used for qRT-PCR analysis of mRNA levels. [file 13287_2022_3032_MOESM2_ESM.pdf]

Table S1. Sequences of primers used for qRT - PCR analysis of mRNA levels

Name	Forward (5'-3')	Reversed (5'-3')
β-actin	GGCTGTATTCCCCTCCATCG	CCAGGTAACAATGCCATG
iNOS	CTCAGCACAGAGGGCTCAAAG	TGCACCCAAACACCAAGGT
IL-10	TTCTTTCAAACAAAGGACCAGC	GCAACCCAAGTAACCCTTAAAG
IL-6	TCTGGAGCCCACCAAGAACGATAG	GTCACCAGCATCAGTCCCAAGAAG
TNFα	GGACTAGCCAGGAGGGAGAACAG	CCAGTGAGTGAAAGGGACAGAAC
IL1β	TCGCAGCAGCACATCAACAAGAG	AGGTCCACGGGAAAGACACAGG
Aif1	CCGAGGAGACGTTCAGCTAC	GACCAGTTGGCCTCTTGTGT
Cx3cr1	AGCTCACGACTGCCTTCTTC	GTCCGGTTGTTCATGGAGTT
H2-D1	CCTCCTCCGTCCACTGACTCTTAC	TTTCCACCTGTGTTTCTCCTTCTCTTC
GBP2	TCAGCAGCACCTTCATCTACAACAG	AGCCCACAAAGTTAGCGGAATCG
Fgf1	GAAAGGCTGGAAGAAAACCATT	CAGAAACAAGATGGCTTTCTGG
Gat3	GCCATTCCACCTGCCTGTCAC	CCACCTCTGCTCTTCGTTTCCAAG
Gat1	GCGTCCCGTGTGATATTGTGTAGG	TCTCCACCGAGCAGGCAGATG
BDNF	CCCATGAAAGAAGTAAACGTCC	CCTTATGGTTTTCTTCGTTGGG
HGF	ACCTACAGGAAAACTACTGTCG	TGCATTCAACTTCTGAACACTG
Sema3a	CATGCTCACGCTATTTTCCTAC	GATGATTATCATGGTGCTGCAA
Sema3b	GAAGACTCCTTATGACCCAAGG	GCTTCGAAAGATGGTAAAGTCC
Grm1	TGAAGTCATCGAAGGCTATGAG	CCTCAGCTTCAGAAAGTAGTCA
C3	GTTTCTGAACACAGCCAAAGAT	GACATCTGTTTGATATTGGGCC
Cxcl1	CAATGAGCTGCGCTGTCAGT	TTGAGGTGAATCCCAGCCAT
Cxcl5	TGATCGCTAATTTGGAGGTGAT	TAGCTTTCTTTTTGTCACTGCC
Ccl7	ACAAAAGATCCCCAAGAGGAAT	TCTTGAAGATAACAGCTTCCCA
S100a9	CACAGTTGGCAACCTTTATGAA	TCATACACTCCTCAAAGCTCAG
Fgf16	GTACCTAGGAATGAATGAGCGA	ATTTCTGGTGTCGTTTAGTCCT
Efna5	ATGTTTTCTGCCCTCACTATGA	ATTCCCATCTCTTGAACCCTTT
Pla2g4e	GCTTGGATTCAGCCTGTGCC	ACCTCATCCTCATGCAGGCC
Tfap2b	CATAGCTCGAGACTCTCTCAAC	CTTGACTCTGGTGGTAGGGTAG
Htr2a	CAATGTGTTTGTCTGGATTGGT	CTGGAGCTGACTAGACTTGTAG
P2y1	CAAGCTGCAGAGATTCATCTTC	GTCATAGCAGGTGACAGTTTTG
P2y2	GTCGTGGCTCTCTATATCTTCC	CGTAGTAATAAACCAACAGCGG
Htr5a	GGAAAATATACAAAGCCGCCAA	CTTGTCTGGAAGGTTACTGTTG
Ntn1	CGGCGCTGCAGATTCAACAT	TAGGCTTGCCCATGTCTCGG
Sema6d	TCCAAACGCCACTCACGACT	GGTGCTCCCGCTTACTGGAT
Hspb1	CTCACAGTGAAGACCAAGGAAG	GAGAGATGTAGCCATGTTCGTC
Efna5	ATGTTTTCTGCCCTCACTATGA	ATTCCCATCTCTTGAACCCTTT
NGF	TCAGACACTCTGGATCTAGACT	CTGTTGTTAATGTTCACCTCGG

Table S1 lists the primer sequences used in the experiments.
