# Supplementary figures and images for: Ectoderm-derived frontal bone mesenchymal stem cells promote traumatic brain injury recovery by alleviating neuroinflammation and glutamate excitotoxicity partially via FGF1
Source: Stem Cell Res Ther. 2022 Jul 26;13:341. doi: 10.1186/s13287-022-03032-6 (PMC9327213; doi:10.1186/s13287-022-03032-6)

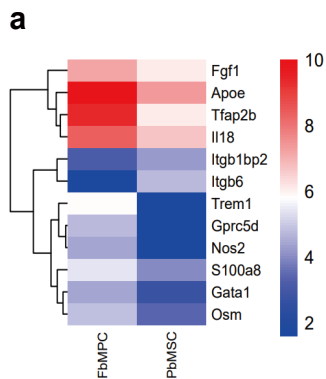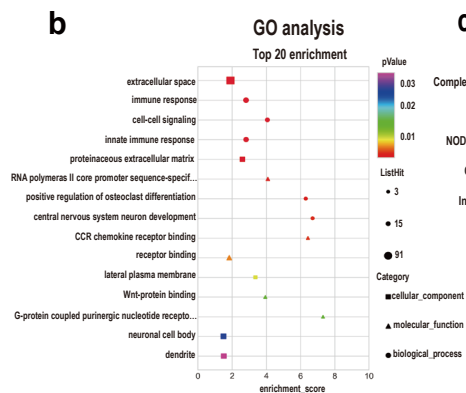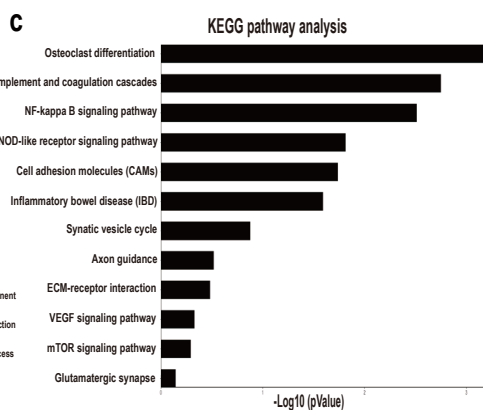

Supplement: Supplementary file 3 — Additional file 3: Affymetrix Clariom D array showed differences between FbMSCs and PbMSCs. [file 13287_2022_3032_MOESM3_ESM.pdf]

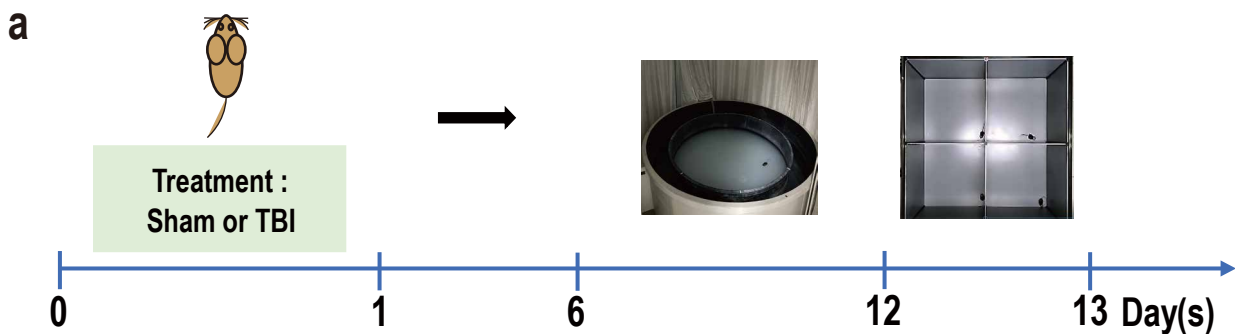

**b** Morris Water Maze Test

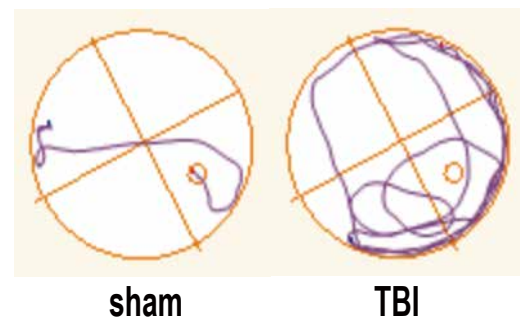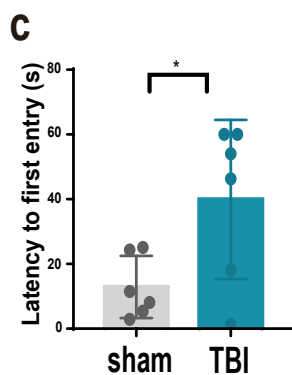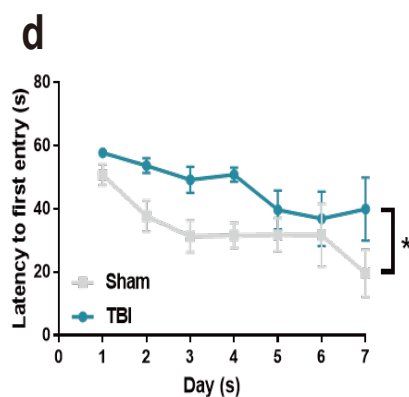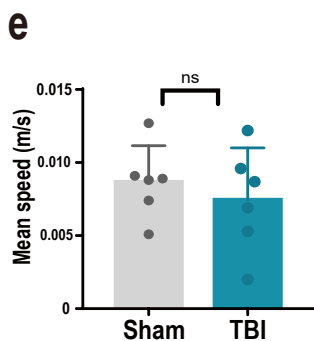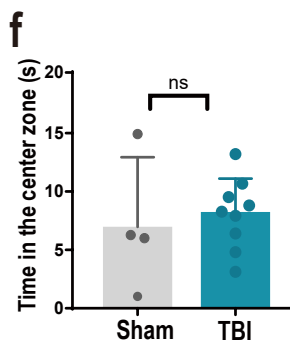

**Open field test**

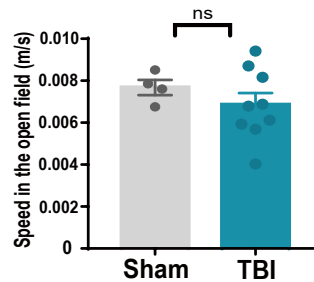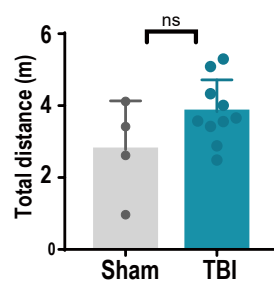

Supplement: Supplementary file 4 — Additional file 4: Traumatic brain injury damages the learning and cognitive ability of mice. [file 13287_2022_3032_MOESM4_ESM.pdf]

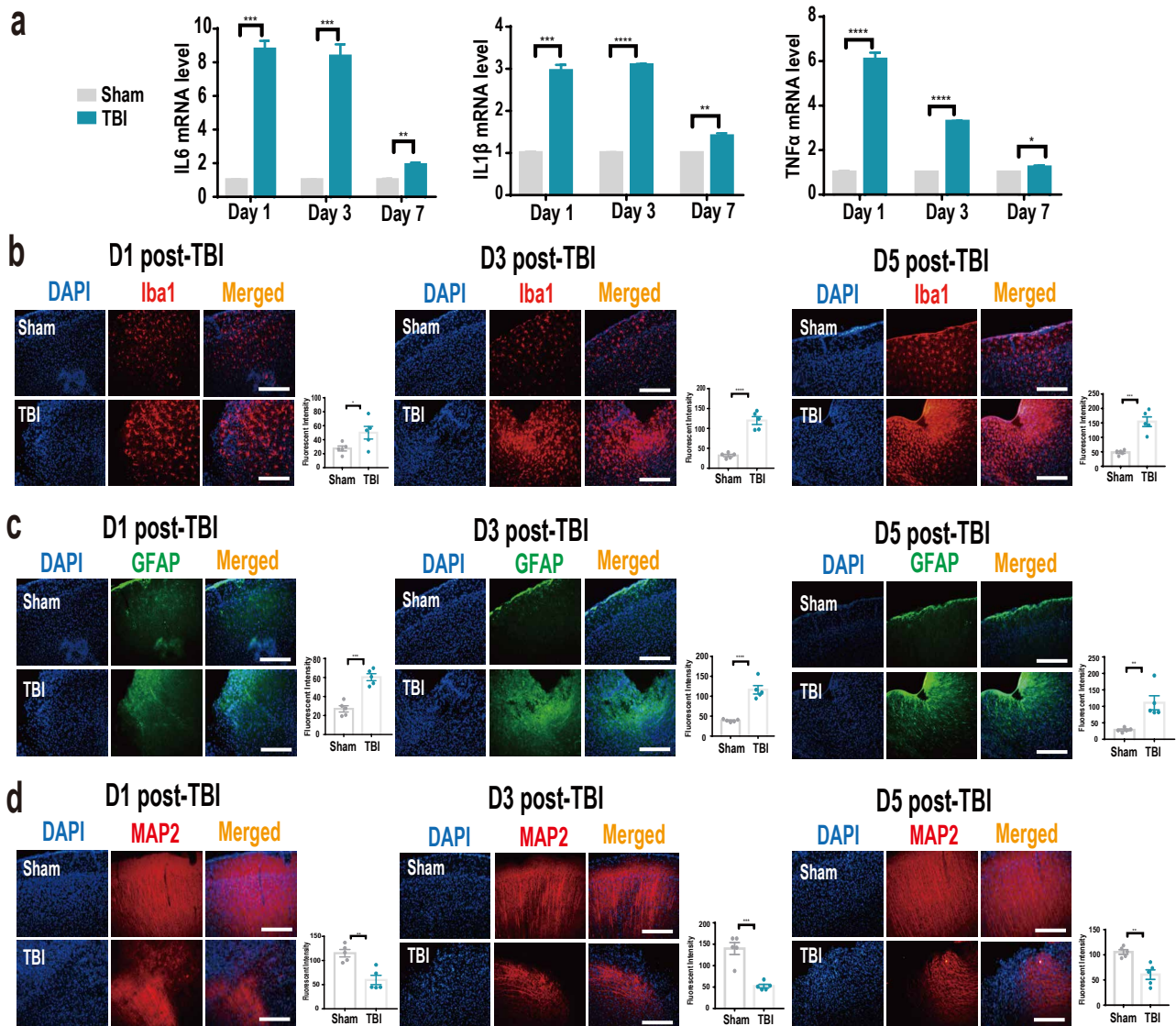

Supplement: Supplementary file 5 — Additional file 5: Adverse changes of brain microenvironment in mice with brain injury. [file 13287_2022_3032_MOESM5_ESM.pdf]
